# Nucleic Acid-Based Therapeutic Approach for Spinal and Bulbar Muscular Atrophy and Related Neurological Disorders

**DOI:** 10.3390/genes13010109

**Published:** 2022-01-05

**Authors:** Tomoki Hirunagi, Kentaro Sahashi, Katherine G. Meilleur, Masahisa Katsuno

**Affiliations:** 1 Department of Neurology, Nagoya University Graduate School of Medicine, 65 Tsurumai-cho, Syowa-ku, Nagoya 466-8550, Japan; hirunagi.tomoki@med.nagoya-u.ac.jp (T.H.); sahashik@med.nagoya-u.ac.jp (K.S.); 2 Research and Clinical Development, Neuromuscular Development Unit, Biogen, 300, Binney Street, Cambridge, MA 02142, USA; katy.meilleur@biogen.com; 3 Department of Clinical Research Education, Nagoya University Graduate School of Medicine, 65 Tsurumai-cho, Syowa-ku, Nagoya 466-8550, Japan

**Keywords:** nucleic acid therapeutics, spinal and bulbar muscular atrophy, polyglutamine disease, motor neuron disease, antisense oligonucleotide, small interference RNA

## Abstract

The recent advances in nucleic acid therapeutics demonstrate the potential to treat hereditary neurological disorders by targeting their causative genes. Spinal and bulbar muscular atrophy (SBMA) is an X-linked and adult-onset neurodegenerative disorder caused by the expansion of trinucleotide cytosine-adenine-guanine repeats, which encodes a polyglutamine tract in the *androgen receptor* gene. SBMA belongs to the family of polyglutamine diseases, in which the use of nucleic acids for silencing a disease-causing gene, such as antisense oligonucleotides and small interfering RNAs, has been intensively studied in animal models and clinical trials. A unique feature of SBMA is that both motor neuron and skeletal muscle pathology contribute to disease manifestations, including progressive muscle weakness and atrophy. As both motor neurons and skeletal muscles can be therapeutic targets in SBMA, nucleic acid-based approaches for other motor neuron diseases and myopathies may further lead to the development of a treatment for SBMA. Here, we review studies of nucleic acid-based therapeutic approaches in SBMA and related neurological disorders and discuss current limitations and perspectives to apply these approaches to patients with SBMA.

## 1. Introduction

Spinal and bulbar muscular atrophy (SBMA) is an X-linked and adult-onset inherited motor neuron disease caused by abnormal cytosine-adenine-guanine (CAG) repeat expansions, which encode polyglutamine (polyQ) tracts in the *androgen receptor* (*AR*) gene [1,2]. Patients with SBMA develop progressive neurological symptoms, including muscle weakness and atrophy, tremor, fasciculation, muscle cramping and dysphasia. A neuropathological hallmark of SBMA is the degeneration of motor neurons in the brain stem and spinal cord [3,4]. However, recent evidence strongly suggests that the disturbance of skeletal muscle also contributes to disease pathogenesis [5,6,7].

SBMA shares its characteristics of three different types of neurological disorders. First, SBMA belongs to polyQ diseases, characterized by intranuclear aggregates of polyQ-expanded proteins in specific neuronal populations. Second, SBMA is one of several motor neuron diseases, a group of disorders that selectively affect motor neurons in the central nervous system (CNS). Third, SBMA also has characteristics typical of myopathies. The remarkable development of nucleic acid therapeutics for these disorders indicates the potential to treat SBMA with nucleic acid-based drugs. In this review, we summarize recent studies of nucleic acid-based therapeutic approaches in SBMA and related neurological disorders and discuss current limitations and perspectives for applying them to patients with SBMA.

## 2. Nucleic Acid-Based Therapeutic Approach for Neurological Disorders

Over the last decade, several nucleic acid therapeutics have been approved for the treatment of genetically inherited disorders [8]. In the treatment of neurological disorders, there are three types of nucleic acid-based therapeutic approaches: antisense oligonucleotides (ASOs)-mediated gene suppression or splicing modulation, RNA interference and adeno-associated virus (AAV) vector-mediated gene therapies [8,9,10] (Table 1). Before reviewing these approaches for SBMA, we provide a brief overview of them in the related disorders, including polyQ diseases, motor neuron diseases and myopathies with a particular emphasis on recent advances. In this review, the word “nucleic acid-based therapeutic approach” includes all nucleic acid-based approaches that modulate gene expression, such as ASOs, small interfering RNAs (siRNAs), microRNAs (miRNAs) and AAV vector-mediated gene therapies.

### 2.1. Polyglutamine Diseases

PolyQ diseases are inherited neurodegenerative disorders caused by abnormal expansions of CAG repeats in causative genes. These diseases include SBMA, Huntington’s disease (HD), dentatorubral-pallidoluysian atrophy, and spinocerebellar ataxia (SCA) types 1, 2, 3, 6, 7, and 17 [11,12]. Patients with polyQ diseases develop progressive neurological symptoms caused by the degeneration of specific neuronal populations.

Reducing polyQ-expanded protein levels is a promising therapeutic strategy in polyQ diseases because toxic polyQ proteins play a central role in disease pathogenesis. The use of ASOs and RNA interference for silencing a disease-causing gene has been intensively studied, which has been reviewed previously [13,14,15,16]. We will briefly provide the recent topics of treatment for HD in this section.

Clinical trials of ASOs targeting *Huntingtin* (*HTT*) mRNA in HD have attracted great attention. A phase 1/2a clinical trial showed that repeated intrathecal administration of an ASO-targeting *HTT* (tominersen) successfully reduced cerebrospinal fluid (CSF) levels of mutant HTT protein [17]. However, a phase 3 trial of tominersen was halted because it failed to show higher clinical efficacy than placebo (NCT03761849). The reason of the failure is unclear; however, several potential explanations have been discussed [18,19]. First, concomitant silencing of wild-type *HTT* by the ASO might compromise the gene function. Second, tominersen may not have efficiently reached the affected regions of the brain. Another possibility is that the timing of the ASO administration was too late to improve neurological symptoms. A mutant allele selective ASO targeting *HTT* SNP is currently undergoing a clinical trial (NCT05032196).

Recently, other therapeutic candidates for the treatment of HD have been reported in preclinical studies. AAV-delivered zinc finger protein transcription factors targeting expanded CAG repeats selectively repressed mutant *HTT* mRNA expression [20]. The study demonstrated intrastriatal injection of the AAV construct ameliorated disease phenotype in three models of HD. A divalent siRNA, which is composed of two fully chemically modified siRNAs connected by a linker, is another new candidate. Intracerebroventricular (ICV) administration of the siRNAs reached multiple brain regions and enabled sustained silencing of *HTT* in mice and non-human primates for at least six and one month(s), respectively [21]. These novel approaches can be therapeutic options, although safety and effectiveness still should be carefully assessed.

### 2.2. Motor Neuron Diseases

Motor neuron diseases (MNDs) are a group of neurological disorders characterized by the degeneration and loss of motor neurons. Patients with MNDs develop progressive muscle weakness and respiratory failure. MNDs generally include SBMA, spinal muscular atrophy (SMA) and amyotrophic lateral sclerosis (ALS). Here, we describe the recent success of nucleic acid therapeutics in SMA and clinical trials in ALS related to *superoxide dismutase 1* (*SOD1*) mutations.

SMA is an autosomal recessive and, typically, childhood-onset MND caused by loss-of-function mutations in the *survival of motor neuron 1* (*SMN1*) gene. *SMN1* mutations result in SMN protein deficiency and cause motor neuron degeneration. There are two innovative drugs approved for SMA: nusinersen and onasemnogene abeparvovec (Table 1). Nusinersen is a fully chemically modified ASO, which targets *SMN2* pre-mRNA. The ASO modulates splicing of *SMN2* to promote exon 7 inclusion and increases SMN protein levels [22]. Two phase 3 clinical trials in infantile- and later-onset SMA demonstrated that infants treated with intrathecal administration of nusinersen had a significantly reduced risk of death or use of permanent ventilation and a better motor-milestone response [23]; treated children with later-onset SMA also had significant and clinically meaningful improvements in motor function [24].

Onasemnogene abeparvovec is an AAV9-mediated gene therapy that restores SMN protein levels [25]. A phase 1 trial showed that, in patients with infantile-onset SMA, a single intravenous injection of onasemnogene abeparvovec resulted in longer survival and superior achievement of motor milestones in comparison with natural history [26]. Two phase 3 clinical trials also showed safety and efficacy of commercial-grade onasemnogene abeparvovec [27,28]; a recent study confirmed widespread induction of SMN protein expression throughout the CNS and peripheral organs [29].

ALS is the most common adult-onset MND, characterized by the pathological involvement of both upper and lower motor neurons. In contrast to SMA, ALS is a heterogeneous disorder, in which genetic and environmental factors may contribute to disease pathogenesis. The most advanced nucleic acid-based approach is to target the *SOD1* gene in ALS due to *SOD1* mutations, which account for up to 2% of all cases [30]. A phase 1–2 clinical trial of intrathecal administration of an ASO targeting *SOD1* mRNA (tofersen) showed a 36% reduction of SOD1 protein concentrations in CSF for the participants who received the highest dose [31]. A recently-completed phase 3 clinical trial (NCT02623699) showed that tofersen did not reach the primary endpoint (change in the Revised Amyotrophic Lateral Sclerosis Functional Rating Scale), although favoring trends were seen in several secondary endpoints and exploratory measures of clinical function, including respiratory function, muscle strength and quality of life [32]. Currently, a phase 3 trial to evaluate the efficacy of tofersen in presymptomatic carriers of an *SOD1* mutation is being conducted (NCT04856982). The effects of nucleic acid therapeutics targeting other genes have also been investigated; phase 1 clinical trials of ASOs targeting *C9orf72*, *ATXN2* and *FUS* have been launched in ALS patients [33]. Furthermore, a recent study showed AAV-mediated delivery of anti-*SOD1* miRNA in two patients with familial ALS with *SOD1* mutations [34]. Although additional studies with a larger number of patients are required, this study showed that AAV-mediated miRNA delivery can be used as a potential treatment for ALS.

### 2.3. Myopathies

Splice-switching ASOs have been approved for the treatment of an inherited myopathy, Duchenne muscular dystrophy (DMD) (Table 1). DMD is the most common type of muscle disorder caused by mutations in the *dystrophin* (*DMD*) gene. Patients with DMD develop progressive muscle weakness between 1 and 6 years of age, accompanied by severe muscle degeneration. Many of them die in their late teens if they are not mechanically ventilated [35]. Splice-switching ASOs induce exon skipping of mutant *DMD* pre-mRNA, restore the reading frame and allow for the production of partially functional, truncated DMD protein [36]. However, clinical trials showed only limited efficacy, and evidence for the clinical effectiveness of these drugs remains to be established [37,38].

Gene silencing nucleic acid therapies have not been approved for the treatment of myopathies. In a mouse model of centronuclear myopathy, systemic administration of ASOs that suppress *dynamin2* expression successfully reversed muscle pathology and extended life span [39]. Based on the success of this study, a phase 1/2 clinical trial of the ASOs has been launched (NCT04743557). In a recent report, ASOs targeting the *DUX4* gene have been tested in a mouse model of facioscapulohumeral muscular dystrophy [40]. The study showed intramuscular injection of the ASOs locally silenced *DUX4* gene expression; however, the effect of systemic administration of the ASOs has not yet been demonstrated.

Gene replacement therapy is potentially an improved strategy for the treatment of myopathies. Currently, three types of AAV-mediated gene therapies are under clinical trials in DMD patients [41]. To pack the large DMD gene in AAV vectors that are limited to an insert size of less than 5 kb, miniaturized transgenes of DMD have been developed [42]. A phase 1/2a clinical trial of intravenous administration of SRP-9001, which delivers micro DMD proteins using an AAV serotype rh74 carrying the tissue specific MHCK7 promoter, showed acceptable tolerability and improvements in serum creatine kinase levels and functional scores [43]. A phase 3 clinical trial of SRP-9001 is currently being conducted (NCT05096221). The other two types of gene therapies, which use different transgenes and AAV serotypes, are also undergoing assessment of safety and efficacy in clinical trials [41].

X-linked myotubular myopathy (XLMTM) is a severe congenital myopathy caused by pathogenic variants in the *myotubularin 1* (*MTM1*) gene [44,45]. AAV-mediated gene therapies have been developed in XLMTM [41]; a phase 1/2 clinical trial of intravenous administration of AT132, which delivers MTM1 proteins using an AAV8 vector, has been conducted (NCT03199469). Unfortunately, four patients treated with AT132 died, and the trial is now on clinical hold. Three patients treated with the higher dose died of complications resulting from liver failure [46]. The cause of death in a patient treated with the lower dose is under investigation [47].

## 3. Nucleic Acid-Based Therapeutic Approach for SBMA

**Figure 1:**
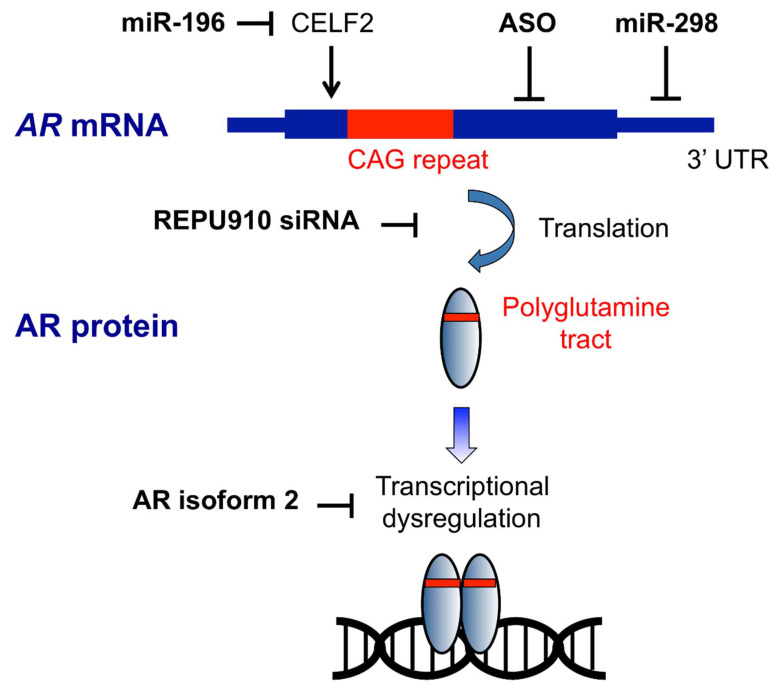
Targets of nucleic acid-based therapeutic strategy in SBMA. ASOs bind to *AR* mRNA and trigger RNase H cleavage and RNA degradation. REPU910 targets expanded CAG repeats, probably through decreasing mRNA stability and translation. Mir-196 silences the *CELF2* gene, which upregulates *AR* expression by enhancing the stability of *AR* mRNA. MiR-298 directly binds to the 3′-untranslated region (UTR) of *AR* mRNA and downregulated the mRNA levels. AR isoform 2 restores transcriptional dysregulation caused by polyglutamine-expanded AR.

Preclinical studies of nucleic acid-based therapeutic approaches for SBMA are summarized in Table 2, and their targets are described in Figure 1. We will describe details of these studies in this section. To date, no clinical trials of nucleic acid therapeutics have been conducted for SBMA.

### 3.1. ASO

ASOs targeting *AR* (AR-ASOs) were first reported in AR113Q knock-in and bacterial artificial chromosome (BAC) transgenic mice (BAC fxAR121) [49]. AR-ASOs are gapmer ASOs that bind to *AR* mRNA and trigger RNase H cleavage and RNA degradation. Subcutaneous administration of AR-ASOs suppressed mutant *AR* expression in the skeletal muscle but not in the CNS. Peripheral suppression of the polyQ-expanded AR rescued deficits in muscle weight, fiber size and grip strength, and extended the life span of both mutant males. By contrast, ICV administration of AR-ASOs did not alter grip strength or survival in BAC fxAR121 mice, despite lowering mutant *AR* expression in the spinal cord. However, it is argued that model-specific phenotypes in AR113Q and BAC fxAR121 mice, such as marked skeletal muscle pathology, might be associated with the limited results in this study.

We explored the therapeutic effect of ICV administration of AR-ASOs in a transgenic model, AR97Q mice, which show SBMA-like neurogenic phenotype [48]. A single ICV administration of the ASOs efficiently suppressed mutant *AR* expression in the CNS but not in the skeletal muscle. The reduction in mutant *AR* in the CNS delayed the onset and progression of motor dysfunction and extended survival with amelioration of histopathology in spinal motor neurons, neuromuscular junctions and skeletal muscle.

These two studies suggest that both motor neuron and skeletal muscle can be therapeutic targets by AR-ASOs in SBMA.

### 3.2. LNP-Delivered siRNA Targeting CAG Expansions

Recently, we reported the therapeutic potential of lipid nanoparticle (LNP)-mediated delivery of siRNA targeting CAG expansions for allele selective suppression of polyQ-expanded proteins in the mouse CNS [50]. The siRNA that was fully complementary to the CAG repeats did not show allele selectivity, whereas the siRNA with a central mismatch and UNA substitutions (REPU910) achieved mutant-allele selective suppression of polyQ-expanded proteins. Although the mechanism of the selectivity has not been fully clarified, these modifications confer structural flexibility to the siRNA, which would compromise AGO2-mediated cleavage of target mRNA and instead provide a mechanism that mimics the action of miRNA, which decrease mRNA stability and translation [54]. ICV administration of LNP delivery of REPU910 suppressed polyQ-expanded proteins in the cerebral cortex of AR97Q mice and an R6/2 transgenic mouse model of HD; however, it did not suppress the expression of wild-type AR with unexpanded CAG repeats in AR24Q mice. In addition, its subcutaneous injection efficiently suppressed polyQ-expanded AR in the skeletal muscle of AR97Q mice. Although the limited biodistribution of the LNP needs to be overcome in future studies, REPU910 has the advantage of selective targeting of CAG expansions, which can be broadly applicable to other polyQ diseases.

### 3.3. AAV-Delivered miRNA

We reported that AAV-mediated delivery of miR-196a ameliorated disease phenotypes and extended survival in AR97Q mice [51]. MiR-196a that is upregulated in the spinal cord of AR97Q mice, as well as SBMA patients, enhanced the decay of *AR* mRNA and reduced the mRNA and protein levels by silencing the *CELF2* gene. CELF2 is found to upregulate *AR* mRNA expression by acting on CUG triplet-repeat sequences and by enhancing the stability of *AR* mRNA.

AAV-delivered miR-298 also ameliorated disease phenotypes in AR97Q mice [52]. MiR-298 directly binds to the 3′-untranslated region of *AR* mRNA and downregulates the *AR* mRNA and protein levels. Interestingly, endogenous miR-298 expression is particularly enriched in the spinal cord, at lower levels in the skeletal muscle, and virtually absent in other tissues.

### 3.4. AAV-Mediated Delivery of AR Isoform 2

A recent study reported AAV9-mediated gene delivery of AR isoform 2, a naturally processed variant encoding a truncated AR lacking the polyQ tract in mouse models of SBMA [53]. Its intravenous administration of AAV9-delivered AR isoform 2 ameliorated disease phenotypes and restored the pathogenic degeneration and transcriptional profile of the skeletal muscle in an AR100Q transgenic mouse model. Furthermore, the treatment did not induce detectable toxicity in BAC fxAR121 mice.

These studies support the therapeutic potential of AAV vector-mediated approaches in SBMA although their effectiveness and potential toxicity, including vector immunogenicity, should be further elucidated.

## 4. Current Limitations and Perspectives

We need to consider that two hurdles exist in applying nucleic acid-based therapeutic approaches to SBMA patients. The first is the issue of drug distribution and duration of action in targeted tissues. AR-ASOs are currently one of the most promising candidates that can be delivered to spinal motor neurons by intrathecal administration. However, gapmer ASOs require repeated administration every few weeks to obtain sufficient, sustained reduction of protein expression of target genes in CSF [17,31], raising concerns about the burden and complications on patients. Systemic administration of AR-ASOs targeting skeletal muscles is another therapeutic option and is less invasive than intrathecal administration; however, it requires much higher doses to suppress mutant *AR* expression, which would cause ASO chemistry-related hepatotoxicity [9]. The optimal dose and frequency of intrathecal or systemic administration of the AR-ASOs remain to be determined for clinical application. Furthermore, although SBMA pathology may be mainly due to gain-of-function toxicity of polyQ-expanded AR [3], we cannot exclude the possibility that the loss of native AR function under the ASO treatment may cause motor and sexual dysfunction. As with AR-ASOs, LNP-delivered siRNAs and AAV vector-based approaches showed therapeutic potential in the mouse models of SBMA. Especially, AAV9-mediated delivery of AR isoform 2 has the advantage of restoring the transcriptional dysregulation without affecting AR native functions. However, there is still limited evidence regarding their pharmacodynamic properties, toxicities and effectiveness, which should be further investigated.

A second problem is how best to evaluate the effect of nucleic acid therapeutics in SBMA patients. Unlike mutant HTT and SOD1 proteins, methods for direct measurement of mutant AR protein have not been established. Reliable assays for detecting mutant AR or other biomarkers in serum or CSF are required for clinical trials. Furthermore, SBMA is relatively slowly progressive compared to HD and ALS, and thus it is difficult to evaluate clinical efficacy of drugs in a short period of time, e.g., one year. Sensitive markers correlated with disease severity, such as biomarkers, imaging and clinical scales, are also necessary for accurate evaluation of the efficacy of the treatment during the timeframe of a clinical trial.

Finally, we discuss the potential applicability of more recent technological advances in SBMA treatment. When targeting mutant AR in motor neurons, newly developed long-acting oligonucleotides, such as divalent siRNAs [21] (described in Section 2.1), may be useful to reduce the frequency of intrathecal administration. A potential limitation of these long-acting oligonucleotides is that unpredictable off-target and adverse effects could persist for a long time in the CNS after a single administration. Bioconjugation strategies can overcome some limitations of ASOs and siRNAs by modulating their pharmacokinetics [55]. A recent report showed DNA/RNA heteroduplex oligonucleotides conjugated to cholesterol or α-tocopherol reached the CNS after systemic administration and silenced target genes in both the CNS and peripheral tissues in rodents [56]. In the skeletal muscle, antitransferrin antibody conjugated to siRNAs showed promise of improved potency [57]. Conjugated oligonucleotides are attractive candidates for SBMA treatment because both motor neurons and skeletal muscles are therapeutic targets. Furthermore, gene-editing approaches, enabled by drug delivery systems using LNP and AAV vector technology, also have the potential to treat neurodegenerative disorders [58]. In addition, repeat-structure-specific DNA ligands, which bind slipped-CAG DNA and contract expanded CAG repeats [59], might be applicable to SBMA.

## 5. Conclusions

In conclusion, we have reviewed nucleic acid-based therapeutic approaches in SBMA and related neurological disorders and discussed current limitations and perspectives to apply these approaches to SBMA patients. Although there are several hurdles to overcome, recent advances in nucleic acid-based technologies can lead to the development of new treatments for SBMA.

## Figures and Tables

**Table 1:** Approved nucleic acid therapeutics for neurological disorders.

Category	Therapeutic Construct	Disease	Target Organ	Administration	Drug Name(s)
ASO	Splice-switching ASO	SMA	CNS	Intrathecal	Nusinersen
		DMD	Skeletal muscle	Intravenous	Eteplirsen, golodirsen, viltolarsen, casimersen
	Gapmer ASO	ATTR ^1^	Liver	Subcutaneous	Inotersen
RNA interference	Liposome-delivered siRNA	ATTR	Liver	Intravenous	Patisiran
Gene therapy	Delivery of SMN	SMA	CNS	Intravenous	Onasemnogene abepavovec

^1^ Hereditary transthyretin amyloidosis.

**Table 2:** Studies of nucleic acid-based therapeutic approaches in SBMA.

Therapeutic Construct	Target	Mouse Model	Administration	Phenotypic Amelioration	Reference
ASO	AR	AR97Q	ICV	+	[48]
	AR	AR113Q BAC fxAR121	Subcutaneous, ICV	AR113Q: subcutaneous +, ICV NA BAC fxAR121: subcutaneous +, ICV −	[49]
LNP-delivered siRNA	CAG repeats	AR97Q AR24Q R6/2	ICV, subcutaneous	NA	[50]
AAV-delivered miRNA	CELF2	AR97Q	Intramuscular	+	[51]
	AR	AR97Q	Intravenous	+	[52]
AAV-mediated delivery of AR isoform 2	AR ^1^	AR100Q BAC fxAR121	Intravenous	AR100Q: + BAC fxAR121: NA	[53]

^1^ Modulating AR transcriptional activity.

## Data Availability

No new data were created or analyzed in this study. Data sharing is not applicable to this article.

## References

[1] Kennedy W.R., Alter M., Sung J.H. (1968). Progressive proximal spinal and bulbar muscular atrophy of late onset. A sex-linked recessive trait. Neurology.

[2] La Spada A.R., Wilson E.M., Lubahn D.B., Harding A.E., Fischbeck K.H. (1991). Androgen receptor gene mutations in X-linked spinal and bulbar muscular atrophy. Nature.

[3] Hashizume A., Fischbeck K.H., Pennuto M., Fratta P., Katsuno M. (2020). Disease mechanism, biomarker and therapeutics for spinal and bulbar muscular atrophy (SBMA). J. Neurol. Neurosurg. Psychiatry.

[4] Arnold F.J., Merry D.E. (2019). Molecular Mechanisms and Therapeutics for SBMA/Kennedy’s Disease. Neurotherapeutics.

[5] Monks D.A., Johansen J.A., Mo K., Rao P., Eagleson B., Yu Z., Lieberman A.P., Breedlove S.M., Jordan C.L. (2007). Overexpression of wild-type androgen receptor in muscle recapitulates polyglutamine disease. Proc. Natl. Acad. Sci. USA.

[6] Palazzolo I., Stack C., Kong L., Musaro A., Adachi H., Katsuno M., Sobue G., Taylor J.P., Sumner C.J., Fischbeck K.H. (2009). Overexpression of IGF-1 in Muscle Attenuates Disease in a Mouse Model of Spinal and Bulbar Muscular Atrophy. Neuron.

[7] Cortes C.J., Ling S.C., Guo L.T., Hung G., Tsunemi T., Ly L., Tokunaga S., Lopez E., Sopher B.L., Bennett C.F. (2014). Muscle expression of mutant androgen receptor accounts for systemic and motor neuron disease phenotypes in spinal and bulbar muscular atrophy. Neuron.

[8] Kulkarni J.A., Witzigmann D., Thomson S.B., Chen S., Leavitt B.R., Cullis P.R., van der Meel R. (2021). The current landscape of nucleic acid therapeutics. Nat. Nanotechnol..

[9] Hammond S.M., Aartsma-Rus A., Alves S., Borgos S.E., Buijsen R.A.M., Collin R.W.J., Covello G., Denti M.A., Desviat L.R., Echevarría L. (2021). Delivery of oligonucleotide-based therapeutics: Challenges and opportunities. EMBO Mol. Med..

[10] Scharner J., Aznarez I. (2021). Clinical Applications of Single-Stranded Oligonucleotides: Current Landscape of Approved and In-Development Therapeutics. Mol. Ther..

[11] Sahashi K., Katsuno M. (2018). Pathogenesis of Polyglutamine Diseases. eLS.

[12] Lieberman A.P., Shakkottai V.G., Albin R.L. (2019). Polyglutamine Repeats in Neurodegenerative Diseases. Annu. Rev. Pathol. Mech. Dis..

[13] Fiszer A., Krzyzosiak W.J. (2014). Oligonucleotide-based strategies to combat polyglutamine diseases. Nucleic Acids Res..

[14] Keiser M.S., Kordasiewicz H.B., McBride J.L. (2016). Gene suppression strategies for dominantly inherited neurodegenerative diseases: Lessons from Huntington’s disease and spinocerebellar ataxia. Hum. Mol. Genet..

[15] Gonzalez-Alegre P. (2019). Recent advances in molecular therapies for neurological disease: Triplet repeat disorders. Hum. Mol. Genet..

[16] Silva A.C., Lobo D.D., Martins I.M., Lopes S.M., Henriques C., Duarte S.P., Dodart J.-C., Nobre R.J., Pereira de Almeida L. (2019). Antisense oligonucleotide therapeutics in neurodegenerative diseases: The case of polyglutamine disorders. Brain.

[17] Tabrizi S.J., Leavitt B.R., Landwehrmeyer G.B., Wild E.J., Saft C., Barker R.A., Blair N.F., Craufurd D., Priller J., Rickards H. (2019). Targeting Huntingtin Expression in Patients with Huntington’s Disease. N. Engl. J. Med..

[18] Kwon D. (2021). Failure of genetic therapies for Huntington’s devastates community. Nature.

[19] Kingwell K. (2021). Double setback for ASO trials in Huntington disease. Nat. Rev. Drug Discov..

[20] Zeitler B., Froelich S., Marlen K., Shivak D.A., Yu Q., Li D., Pearl J.R., Miller J.C., Zhang L., Paschon D.E. (2019). Allele-selective transcriptional repression of mutant HTT for the treatment of Huntington’s disease. Nat. Med..

[21] Alterman J.F., Godinho B.M.D.C., Hassler M.R., Ferguson C.M., Echeverria D., Sapp E., Haraszti R.A., Coles A.H., Conroy F., Miller R. (2019). A divalent siRNA chemical scaffold for potent and sustained modulation of gene expression throughout the central nervous system. Nat. Biotechnol..

[22] Chiriboga C.A., Swoboda K.J., Darras B.T., Iannaccone S.T., Montes J., De Vivo D.C., Norris D.A., Bennett C.F., Bishop K.M. (2016). Results from a phase 1 study of nusinersen (ISIS-SMN Rx) in children with spinal muscular atrophy. Neurology.

[23] Finkel R.S., Mercuri E., Darras B.T., Connolly A.M., Kuntz N.L., Kirschner J., Chiriboga C.A., Saito K., Servais L., Tizzano E. (2017). Nusinersen versus Sham Control in Infantile-Onset Spinal Muscular Atrophy. N. Engl. J. Med..

[24] Mercuri E., Darras B.T., Chiriboga C.A., Day J.W., Campbell C., Connolly A.M., Iannaccone S.T., Kirschner J., Kuntz N.L., Saito K. (2018). Nusinersen versus Sham Control in Later-Onset Spinal Muscular Atrophy. N. Engl. J. Med..

[25] Foust K.D., Wang X., McGovern V.L., Braun L., Bevan A.K., Haidet A.M., Le T.T., Morales P.R., Rich M.M., Burghes A.H.M. (2010). Rescue of the spinal muscular atrophy phenotype in a mouse model by early postnatal delivery of SMN. Nat. Biotechnol..

[26] Mendell J.R., Al-Zaidy S., Shell R., Arnold W.D., Rodino-Klapac L.R., Prior T.W., Lowes L., Alfano L., Berry K., Church K. (2017). Single-Dose Gene-Replacement Therapy for Spinal Muscular Atrophy. N. Engl. J. Med..

[27] Day J.W., Finkel R.S., Chiriboga C.A., Connolly A.M., Crawford T.O., Darras B.T., Iannaccone S.T., Kuntz N.L., Peña L.D.M., Shieh P.B. (2021). Onasemnogene abeparvovec gene therapy for symptomatic infantile-onset spinal muscular atrophy in patients with two copies of SMN2 (STR1VE): An open-label, single-arm, multicentre, phase 3 trial. Lancet Neurol..

[28] Mercuri E., Muntoni F., Baranello G., Masson R., Boespflug-Tanguy O., Bruno C., Corti S., Daron A., Deconinck N., Servais L. (2021). Onasemnogene abeparvovec gene therapy for symptomatic infantile-onset spinal muscular atrophy type 1 (STR1VE-EU): An open-label, single-arm, multicentre, phase 3 trial. Lancet Neurol..

[29] Thomsen G., Burghes A.H.M., Hsieh C., Do J., Chu B.T.T., Perry S., Barkho B., Kaufmann P., Sproule D.M., Feltner D.E. (2021). Biodistribution of onasemnogene abeparvovec DNA, mRNA and SMN protein in human tissue. Nat. Med..

[30] Zou Z.Y., Zhou Z.R., Che C.H., Liu C.Y., He R.L., Huang H.P. (2017). Genetic epidemiology of amyotrophic lateral sclerosis: A systematic review and meta-analysis. J. Neurol. Neurosurg. Psychiatry.

[31] Miller T., Cudkowicz M., Shaw P.J., Andersen P.M., Atassi N., Bucelli R.C., Genge A., Glass J., Ladha S., Ludolph A.L. (2020). Phase 1–2 Trial of Antisense Oligonucleotide Tofersen for SOD1 ALS. N. Engl. J. Med..

[32] Biogen Announces Topline Results from the Tofersen Phase 3 Study and Its Open-Label Extension in SOD1-ALS. https://investors.biogen.com/news-releases/news-release-details/biogen-announces-topline-results-tofersen-phase-3-study-and-its.

[33] Amado D.A., Davidson B.L. (2021). Gene therapy for ALS: A review. Mol. Ther..

[34] Mueller C., Berry J.D., McKenna-Yasek D.M., Gernoux G., Owegi M.A., Pothier L.M., Douthwright C.L., Gelevski D., Luppino S.D., Blackwood M. (2020). SOD1 Suppression with Adeno-Associated Virus and MicroRNA in Familial ALS. N. Engl. J. Med..

[35] Eagle M., Baudouin S.V., Chandler C., Giddings D.R., Bullock R., Bushby K. (2002). Survival in Duchenne muscular dystrophy: Improvements in life expectancy since 1967 and the impact of home nocturnal ventilation. Neuromuscul. Disord..

[36] Mitrpant C., Adams A.M., Meloni P.L., Muntoni F., Fletcher S., Wilton S.D. (2009). Rational design of antisense oligomers to induce dystrophin exon skipping. Mol. Ther..

[37] Stein C.A. (2016). Eteplirsen approved for duchenne muscular dystrophy: The FDA faces a difficult choice. Mol. Ther..

[38] Aartsma-Rus A., Krieg A.M. (2017). FDA Approves Eteplirsen for Duchenne Muscular Dystrophy: The Next Chapter in the Eteplirsen Saga. Nucleic Acid Ther..

[39] Tasfaout H., Buono S., Guo S., Kretz C., Messaddeq N., Booten S., Greenlee S., Monia B.P., Cowling B.S., Laporte J. (2017). Antisense oligonucleotide-mediated Dnm2 knockdown prevents and reverts myotubular myopathy in mice. Nat. Commun..

[40] Lim K.R.Q., Maruyama R., Echigoya Y., Nguyen Q., Zhang A., Khawaja H., Chandra S.S., Jones T., Jones P., Chen Y.W. (2020). Inhibition of DUX4 expression with antisense LNA gapmers as a therapy for facioscapulohumeral muscular dystrophy. Proc. Natl. Acad. Sci. USA.

[41] Mendell J.R., Al-Zaidy S.A., Rodino-Klapac L.R., Goodspeed K., Gray S.J., Kay C.N., Boye S.L., Boye S.E., George L.A., Salabarria S. (2021). Current Clinical Applications of In Vivo Gene Therapy with AAVs. Mol. Ther..

[42] Asher D.R., Thapa K., Dharia S.D., Khan N., Potter R.A., Rodino-Klapac L.R., Mendell J.R. (2020). Clinical development on the frontier: Gene therapy for duchenne muscular dystrophy. Expert Opin. Biol. Ther..

[43] Mendell J.R., Sahenk Z., Lehman K., Nease C., Lowes L.P., Miller N.F., Iammarino M.A., Alfano L.N., Nicholl A., Al-Zaidy S. (2020). Assessment of Systemic Delivery of rAAVrh74.MHCK7.micro-dystrophin in Children with Duchenne Muscular Dystrophy: A Nonrandomized Controlled Trial. JAMA Neurol..

[44] Laporte J., Hu L.J., Kretz C., Mandel J.L., Kioschis P., Coy J.F., Klauck S.M., Poustka A., Dahl N. (1996). A gene mutated in X-linked myotubular myopathy defines a new putative tyrosine phosphatase family conserved in yeast. Nat. Genet..

[45] Beggs A.H., Byrne B.J., De Chastonay S., Haselkorn T., Hughes I., James E.S., Kuntz N.L., Simon J., Swanson L.C., Yang M.L. (2018). A multicenter, retrospective medical record review of X-linked myotubular myopathy: The recensus study. Muscle Nerve.

[46] Philippidis A. (2021). Fourth Boy Dies in Clinical Trial of Astellas’ AT132. Hum. Gene Ther..

[47] Astellas Reports Update to September 1 Announcement on the ASPIRO Clinical Trial of AT132 in Patients with X-Linked Myotubular Myopathy. https://www.astellas.com/en/news/17161.

[48] Sahashi K., Katsuno M., Hung G., Adachi H., Kondo N., Nakatsuji H., Tohnai G., Iida M., Bennett F.F., Sobue G. (2015). Silencing neuronal mutant androgen receptor in a mouse model of spinal and bulbar muscular atrophy. Hum. Mol. Genet..

[49] Lieberman A.P., Yu Z., Murray S., Peralta R., Low A., Guo S., Yu X.X., Cortes C.J., Bennett C.F., Monia B.P. (2014). Peripheral Androg en Receptor Gene Suppression Rescues Disease in Mouse Models of Spinal and Bulbar Muscular Atrophy. Cell Rep..

[50] Hirunagi T., Sahashi K., Tachikawa K., Leu A.I., Nguyen M., Mukthavaram R., Karmali P.P., Chivukula P., Tohnai G., Iida M. (2021). Selective suppression of polyglutamine-expanded protein by lipid nanoparticle-delivered siRNA targeting CAG expansions in the mouse CNS. Mol. Ther. Nucleic Acids.

[51] Miyazaki Y., Adachi H., Katsuno M., Minamiyama M., Jiang Y.-M., Huang Z., Doi H., Matsumoto S., Kondo N., Iida M. (2012). Viral delivery of miR-196a ameliorates the SBMA phenotype via the silencing of CELF2. Nat. Med..

[52] Pourshafie N., Lee P.R., Chen K.L., Harmison G.G., Bott L.C., Katsuno M., Sobue G., Burnett B.G., Fischbeck K.H., Rinaldi C. (2016). MIR-298 counteracts mutant androgen receptor toxicity in spinal and bulbar muscular atrophy. Mol. Ther..

[53] Lim W.F., Forouhan M., Roberts T.C., Dabney J., Ellerington R., Speciale A.A., Manzano R., Lieto M., Sangha G., Banerjee S. (2021). Gene therapy with AR isoform 2 rescues spinal and bulbar muscular atrophy phenotype by modulating AR transcriptional activity. Sci. Adv..

[54] Hu J., Liu J., Corey D.R. (2010). Allele-selective inhibition of huntingtin expression by switching to an miRNA-like RNAi mechanism. Chem. Biol..

[55] Benizri S., Gissot A., Martin A., Vialet B., Grinstaff M.W., Barthélémy P. (2019). Bioconjugated Oligonucleotides: Recent Developments and Therapeutic Applications. Bioconjug. Chem..

[56] Nagata T., Dwyer C.A., Yoshida-Tanaka K., Ihara K., Ohyagi M., Kaburagi H., Miyata H., Ebihara S., Yoshioka K., Ishii T. (2021). Cholesterol-functionalized DNA/RNA heteroduplexes cross the blood–brain barrier and knock down genes in the rodent CNS. Nat. Biotechnol..

[57] Sugo T., Terada M., Oikawa T., Miyata K., Nishimura S., Kenjo E., Ogasawara-Shimizu M., Makita Y., Imaichi S., Murata S. (2016). Development of antibody-siRNA conjugate targeted to cardiac and skeletal muscles. J. Control. Release.

[58] Zhu X., Zhang Y., Yang X., Hao C., Duan H. (2021). Gene Therapy for Neurodegenerative Disease: Clinical Potential and Directions. Front. Mol. Neurosci..

[59] Nakamori M., Panigrahi G.B., Lanni S., Gall-Duncan T., Hayakawa H., Tanaka H., Luo J., Otabe T., Li J., Sakata A. (2020). A slipped-CAG DNA-binding small molecule induces trinucleotide-repeat contractions in vivo. Nat. Genet..

